# Promoter Optimization Circumvents Bcl-2 Transgene-Mediated Suppression of Lentiviral Vector Production

**DOI:** 10.3390/biom13091397

**Published:** 2023-09-16

**Authors:** Cindy Y. Kok, Lauren M. MacLean, Renuka Rao, Shinya Tsurusaki, Eddy Kizana

**Affiliations:** 1 Centre for Heart Research, The Westmead Institute for Medical Research, Westmead, NSW 2145, Australia; c.kok@sydney.edu.au (C.Y.K.); lmac6788@uni.sydney.edu.au (L.M.M.); renuka.rao@sydney.edu.au (R.R.); shinya.tsurusaki@sydney.edu.au (S.T.); 2 Westmead Clinical School, The University of Sydney, Westmead, NSW 2145, Australia; 3 Department of Cardiology, Westmead Hospital, Westmead, NSW 2145, Australia

**Keywords:** lentivirus, promoter, apoptosis, gene therapy, promoter, cardiotoxicity

## Abstract

Lentiviral vectors are a robust gene delivery tool for inducing transgene expression in a variety of cells. They are well suited to facilitate the testing of therapeutic candidate genes in vitro, due to relative ease of packaging and ability to transduce dividing and non-dividing cells. Our goal was to identify a gene that could be delivered to the heart to protect against cancer-therapy-induced cardiotoxicity. We sought to generate a lentivirus construct with a ubiquitous CMV promoter driving expression of B-cell lymphocyte/leukemia 2 gene (*Bcl-2*), a potent anti-apoptotic gene. Contrary to our aim, overexpression of Bcl-2 induced cell death in the producer HEK293T cells, resulting in failure to produce usable vector titre. This was circumvented by exchanging the CMV promoter to the cardiac-specific NCX1 promoter, leading to the successful production of a lentiviral vector which could induce cardioprotective expression of Bcl-2. In conclusion, reduced expression of Bcl-2 driven by a weaker promoter improved vector yield, and led to the production of functional cardioprotective Bcl-2 in primary cardiomyocytes.

## 1. Introduction

Doxorubicin is an anti-cancer drug with high efficacy against various adult and paediatric cancers. The therapeutic utility of doxorubicin is significantly limited by the development of dose-dependent cardiac toxicity. In the 1970s, 4–36% of patients who received a doxorubicin dose of 500–600 mg/m^2^ progressed to heart failure [1], though this incidence has reduced with lower modern dosing regimes [2]. In severe cases, cardiotoxicity presents as severe heart failure which is largely irreversible and carries a poor prognosis. Cardiomyocyte death by apoptosis is a principal mechanism of doxorubicin-induced cardiotoxicity [3]. The B-cell lymphocyte/leukemia 2 gene (*Bcl-2*) is a potent inhibitor of apoptosis and thereby a promising protective agent against doxorubicin and other cancer-therapy-induced cardiotoxicity.

Bcl-2 has been shown to be protective against doxorubicin-induced injury in a variety of cell types. A study conducted on human liver cancer cells (HCC) investigated the protective ability of Bcl-2 in a recombinant vector against doxorubicin-mediated cell death (0.01, 0.1 and 1μg/mL doxorubicin) [4]. High levels of Bcl-2 expression were shown to prevent doxorubicin-induced apoptosis in HCC cell lines by converting doxorubicin from a cytotoxic to a cytostatic drug [4]. Similar results have been observed in leukemia [5,6,7], bladder cancer [8], and neuroblastoma cell lines [9].

We sought to determine whether we could achieve protection against doxorubicin-mediated cardiotoxicity using Bcl-2 gene therapy. To test candidate genes for cardioprotection in vitro, lentiviruses are ideal as they have a large packaging capacity and are able to transduce proliferating and quiescent cells. Unexpectedly, lentiviral vector could not be produced when Bcl-2 expression was driven by the CMV promoter. We therefore investigated the cause of cell death, and circumvented vector production issues by using a tissue-specific promoter to lower the level of transgene expression during production. Subsequently vector titre was improved, leading to production of functional and cardioprotective Bcl-2 expression in doxorubicin-treated cardiomyocytes.

## 2. Materials and Methods

### 2.1. Maintenance of HEK293T Cells

The HEK293T cells (ATCC, CRL-3216^TM^, Manassas, VA, USA) were maintained in Dulbecco’s Modified Eagle Medium (DMEM) (Lonza, #12-604F, Basel, Switzerland) supplemented with 10% FBS (*v*/*v*) (Gibco, #1099-141, NSW, Australia) and 1% L-glutamine (*v*/*v*) (Sigma-Aldrich, #G7513-100ML, St. Louis, MO, USA) at 37 °C in a humidified atmosphere containing 5% (*v*/*v*) CO_2_ cells.

### 2.2. Molecular Cloning and Lentiviral Production

The p.Bcl-2 plasmid was a gift from Prof David Vaux (Walter and Eliza Hall Institute of Medical Research). The *Bcl-2* cDNA was cloned into the lentiviral vector plasmid pRRLsin18.cPPT.CMV.GFP.Wpre (pPPT.CMV.GFP, Inder Verma, The Salk Institute for Biological Studies, La Jolla, CA, USA) after removal of GFP. The new vector was named ppt.CMV.Bcl-2. Lentiviral vectors were produced in HEK293T cells via calcium phosphate precipitation using the expression construct (ppt.CMV.GFP, ppt.CMV.Bcl-2, ppt.NCX1.GFP, ppt.NCX1.Bcl-2) and pMDLgpRRE, pRSVREV, pMD2VSVG packaging plasmids as previously described [10]. Vector-containing supernatant was collected at 48 and 72 h after transfection, then filtered and concentrated by ultrafiltration (100,000 MWCO PES, sartorius, #VS2042, Gottingen, Germany). Concentrated virus (LV.CMV.GFP or Bcl-2 and LV.NCX1.GFP or Bcl-2) was aliquoted and stored at −80 °C. Transduction titre was assigned to concentrated supernatant by assessing transgene expression in HEK293T cells using a limiting dilution assay in the presence of polybrene 8 μg/mL (Sigma-Aldrich, #H9268-10G, St. Louis, MO, USA) four days after transduction. For transduction experiments, concentrated vector stock was used at the indicated multiplicity of transduction (MOT) in the presence of polybrene 8 mg/mL. Vector was applied overnight and media changed the following morning. Cells were then harvested for various assays on day 4 post-transduction. 

### 2.3. Amplification of Bcl-2 and GAPDH Transcript by Quantitative PCR

RNA was extracted from transfected HEK293T (6 well plate, 2.5 mg of DNA per well) and transduced NRVM cells (24 well plate, MOT 5) using the ISOLATE II RNA Mini Kit (Bioline, #BIO-52072, Memphis, TN, USA) then quantified using a NanoDrop™ 2000 Spectrophotometer (Thermo Fisher Scientific, #ND2000, Waltham, MA, USA). For cDNA synthesis, Random Primers (500 ng/μL) were added to the extracted RNA (1 μg), followed by incubation at 70 °C for 5 min. M-MLV Reverse Transcriptase Buffer (5X), M-MLV Reverse Transcriptase (Promega, #M1701, Madison, WI, USA), 10 mM dNTPs (New Engliand Biolabs, #N0447S, Ipswich, MA, USA) and RNasin^®^ Ribonuclease Inhibitor (Promega, #N2111, Madison, WI, USA) were then added to the reaction mixture, followed by incubation under the following conditions: 25 °C for 10 min, 37 °C for 1 h then 70 °C for 15 min. A relative qPCR was performed on the synthesized cDNA using the SensiFAST™ SYBR^®^ No-ROX Kit (Bioline, #BIO-98020, Memphis, TN, USA) on the CFX96™ Real-Time System C1000 Touch™ Thermal Cycler (Biorad, #1855196, Hercules, CA, USA) to amplify Bcl-2 and GAPDH (Table 1).

### 2.4. Evaluation of Bcl-2 Mediated Toxicity in HEK293T

HEK293T cells were seeded at 5 × 10^5^ cells/well in 24-well plates. Twenty-four hours later (D1), cells were transfected with lentiviral plasmids (ppt.CMV.GFP, ppt.CMV.Bcl-2, ppt.NCX1.GFP or ppt.NCX1.Bcl-2) at 0, 50, 100, 200 and 500 ng. Media were changed on D2, followed by MTT assay on day 4. Cell viability was assessed on D3 using the CellTiter 96^®^ Non-Radioactive Cell Proliferation Assay (Promega, #G4000, Madison, WI, USA), hereafter abbreviated to “MTT assay”, according to the manufacturer’s instructions. 

### 2.5. Neonatal Rat Ventricular Myocyte Isolation

NRVMs were isolated from day 3 (D3) neonatal Wistar rats via enzymatic dissociation of ventricles using trypsin and collagenase as previously described [11]. Isolated NRVMs were seeded at 2–4 × 10^5^ cells/well in 24-well plates. Twenty-four hours later, cells were washed with DPBS (Lonza, #12001-664, Basel, Switzerland). On D2 after cell plating, FBS was reduced to 2% and media were changed on cultures every second day.

### 2.6. Doxorubicin Injury of NRVMs

To investigate the effect of Bcl-2 overexpression, LV.NCX1.GFP- or LV.NCX1.Bcl-2-transduced NRVMs in 24-well plates were injured at D4 post-transduction with a dose titration of doxorubicin (Adriamycin, 3.7 mM solution). After 48 h, cell viability was assessed using the MTT assay, according to the manufacturer’s instructions. Cell viability was expressed as a percentage of the un-injured control (0 μM doxorubicin) within each treatment group.

### 2.7. Statistical Analysis and Software

Depending on the data, different statistical analyses detailed in the figure legends were performed using GraphPad Prism (GraphPad Software version 9.5.1, La Jolla, CA, USA). For all used tests, significance was represented as follows: * *p* < 0.05, ** *p* < 0.01, *** *p* < 0.001, and **** *p* < 0.0001. SnapGene software (Dotmatics, San Diego, CA, USA) version 5.1.3 was used to design cloning strategies to generate the Bcl-2 lentiviral construct.

## 3. Results

**Figure 1:**
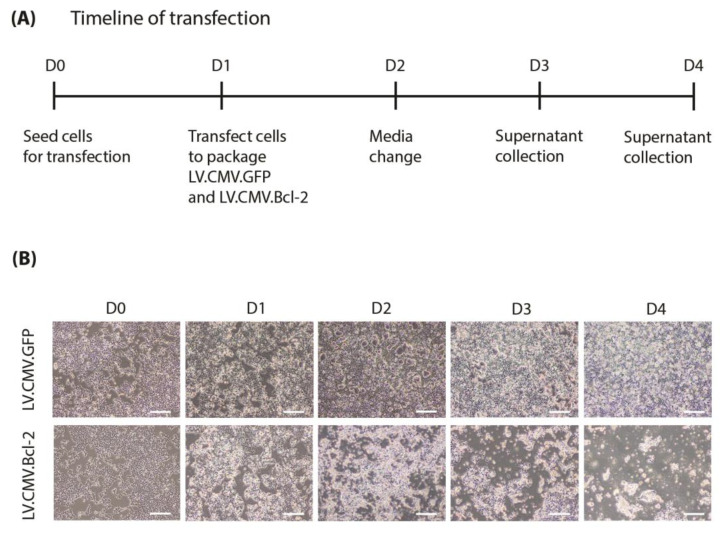
Vector titre was reduced by Bcl-2 transgene expression during lentivirus packaging. (**A**) Timeline of transfection protocol for lentivirus packaging. HEK293T cells were transfected in T150 flasks by calcium phosphate precipitation and monitored after transfection on day 1. (**B**) Cells were imaged daily from D0 to D4 (scale bar = 200 mm).

To produce lentivirus, HEK293T cells were transfected with the lentiviral packaging plasmids and expression construct on D1, then virus-containing media were collected at D3 and D4 (Figure 1A). An unexpected trend of increasing cell death over the course of lentivirus production was observed in cells transfected with plasmid mix containing ppt.CMV.Bcl-2 (Figure 1B). These cells showed reduced confluence relative to the ppt.CMV.GFP control, with dying cells rounded and floating in the culture media. 

**Figure 2:**
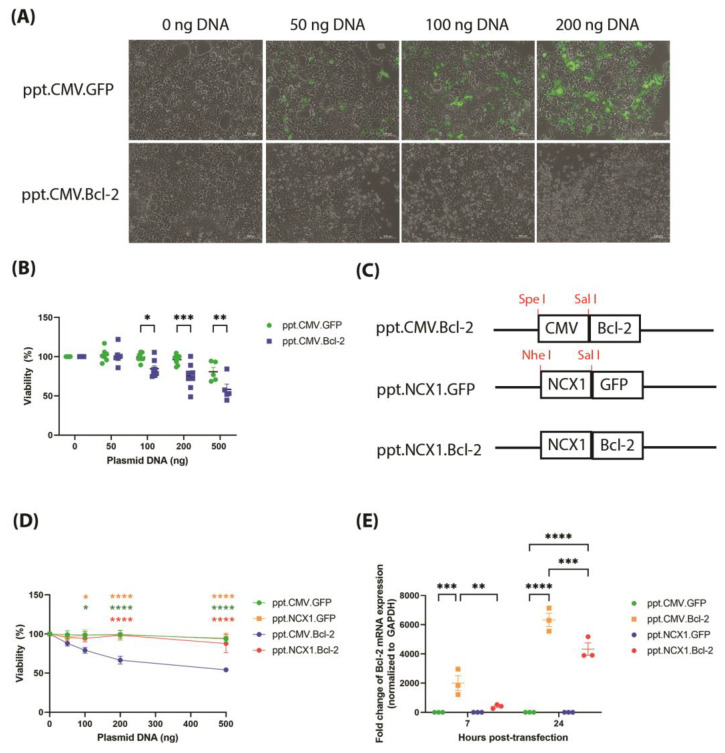
Bcl-2-mediated toxicity in HEK293T cells was ameliorated by changing the CMV promoter to the cardiac specific NCX1 promoter. (**A**) HEK293T cells were transfected with 0–200 ng of the lentiviral plasmid construct (ppt.CMV.GFP or ppt.CMV.Bcl-2) and imaged prior to harvest 48 h post-transfection (scale bar = 100 mm). Merged image of green fluorescence and brightfield is shown. (**B**) Viability of transfected cells assessed by MTT assay, with data expressed as a percentage of the non-transfected control for each treatment group (ppt.CMV.GFP or ppt.CMV.Bcl-2, *n* = 5–8 per group). Each group was compared to ppt.CMV.GFP, using an ordinary two-way ANOVA, and the difference calculated with Sidak’s multiple comparison test. (**C**) Schematic showing cloning strategy for generating the ppt.NCX1.Bcl-2, by first removing the CMV promoter from ppt.CMV.Bcl-2 using Spe I and Sal I double digestion, then replacing with the NCX1 promoter which was excised from ppt.NCX1.GFP using Nhe I and Sal I double digest. (**D**) Viability of transfected cells assessed by MTT assay, with data expressed as a percentage of the non-transfected control for each treatment group (ppt.CMV.GFP, ppt.CMV.Bcl-2, ppt.NCX1.GFP or ppt.NCX1.Bcl-2, *n* = 6 per group). Difference of all groups was compared to ppt.CMV.Bcl-2, using an ordinary two-way ANOVA, and the difference calculated with Sidak’s multiple comparison test. (**E**) Bcl-2 expression in transfected cells measured by qPCR 7 hr and 24 hr post transfection (*n* = 3 per group). Data are normalised to GAPDH and expressed as fold change over ppt.CMV.GFP. All data are mean ± SEM. Statistical significance of differences was calculated using an ordinary two-way ANOVA, and Tukey’s multiple comparison test (* *p* ≤ 0.05, ** *p* ≤ 0.01, *** *p* ≤ 0.001, **** *p* ≤ 0.0001).

We further investigated this phenomenon by transfecting cells with ppt.CMV.Bcl-2 in the absence of the lentivirus packaging plasmids, and observed from cell morphology that Bcl-2-mediated toxicity was dose-dependent (Figure 2A). A significant reduction in viability was observed in cells transfected with ≥100 ng of ppt.CMV.Bcl-2, relative to the ppt.CMV.GFP control (Figure 2B). To inhibit Bcl-2 expression during vector packaging, the ubiquitous CMV promoter was changed to the cardiac-specific NCX1 promoter [12]. The resulting vector was called ppt.NCX1.Bcl-2 (Figure 2C). A comparison of the vectors showed no change in viability in HEK293T cells transfected with GFP plasmids driven by either CMV or NCX1. Encouragingly, cell death was ameliorated in the cells transfected with ppt.NCX1.Bcl-2, relative to the ppt.CMV.Bcl-2 control (Figure 2D). Further dissection of acute Bcl-2 expression post-transfection showed that NCX1 was a weaker promoter compared to CMV. Significantly weaker Bcl-2 expression was observed in ppt.NCX1.Bcl-2-transfected cells relative to the CMV control at both 7 hr and 24 hr timepoints (Figure 2E).

**Figure 3:**
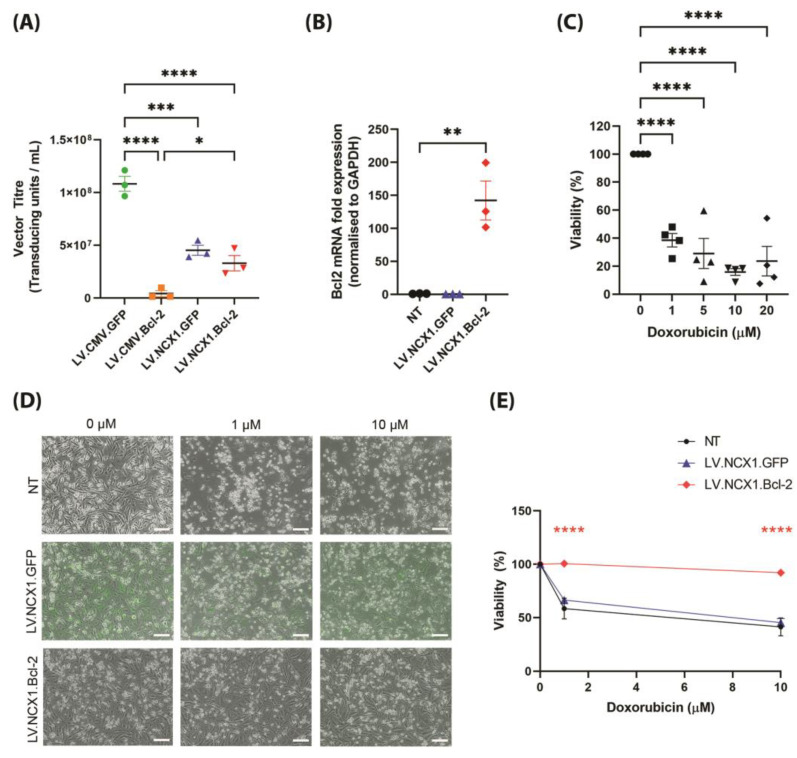
Reduced Bcl-2-mediated toxicity improved packaging efficiency, and resulted in production of function Bcl-2-encoding lentiviral vector. (**A**) Titre of lentiviral vectors quantified by qPCR from genomic DNA of transduced HEK293T cells. Statistical significance of differences was calculated using an ordinary one-way ANOVA, and the difference was calculated with Tukey’s multiple comparison test. (**B**) Bcl-2 expression in transfected NRVMs measured by qPCR (*n* = 3 per group). Data are normalised to GAPDH and expressed as fold change over NT. Statistical significance of differences was calculated using an ordinary one-way ANOVA, and the difference was calculated with Dunnett’s comparison. (**C**) Titration of doxorubicin in NRVMs 0–20 μM, with viability of cells assessed by MTT assay. Data expressed as a percentage of the untreated control. Statistical significance of differences was calculated using an ordinary one-way ANOVA, and the difference was calculated with Dunnett’s comparison. (**D**) Transduced cells in 24-well plates were treated with doxorubicin for 48 h at day 4 post transduction (*n* = 3). Fluorescence images showing merged GFP fluorescence and brightfield in live cells (scale bar = 100 mm). (**E**) Viability was then assessed using the MTT assay, with viability expressed as a percentage of the non-injured control (0 µM doxorubicin) of each group. Statistical significance of differences was calculated using a two-way ANOVA with Dunnett’s multiple comparison test (* *p* ≤ 0.05, ** *p* ≤ 0.01, *** *p* ≤ 0.001, **** *p* ≤ 0.0001). All data are mean ± SEM.

We next investigated whether reducing Bcl-2 expression enabled improved lentiviral vector yields. Interestingly, both vectors containing the NCX1 promoter showed significantly fewer transducing units relative to the LV.CMV.GFP control (Figure 3A). However, LV.NCX1.Bcl-2 showed improved vector production compared to LV.CMV.Bcl-2, and was not different to LV.NCX1.GFP. The resulting vector was shown to be active in NRVMs, with a 142.3-fold increase in Bcl-2 expression compared to the NT control (Figure 3B). We also sought to validate the anti-apoptotic function of the Bcl-2 protein by using an in vitro model of doxorubicin cardiotoxicity. The dose range required to induce cell death was determined in NRVMs (Figure 3C). This doxorubicin dose range was then applied to NRVMs transduced with LV.NCX1.Bcl-2 or LV.NCX1.GFP at day 4 post transduction. While survival was unchanged in GFP-expressing cells relative to the NT control, Bcl-2 overexpression successfully rescued NRVMs from doxorubicin-mediated toxicity at every dose tested (Figure 3D).

## 4. Discussion

Doxorubicin has been shown to reduce Bcl-2, a well-known suppressor of apoptosis, which renders cardiomyocytes vulnerable to cell death [13]. Augmenting Bcl-2 expression with Berberine has been shown as one indirect method of inducing cardioprotection [14]. In this work, we sought to explore the use of gene therapy to induce the overexpression of Bcl-2 to protect against doxorubicin cardiotoxicity. Unexpectedly, we observed that a lentiviral vector encoding Bcl-2 could not be produced due to toxicity in the producer cells. 

When Bcl-2 is transiently expressed in HEK293 cells, it has been shown to become pro-apoptotic [15,16,17]. This could be due to the magnitude of overexpression, as well as subcellular localization of the transgene [18]. Since producer cell toxicity is transgene-specific, toxicity would occur independent of the vector system used for packaging [19]. Transiently overexpressed Bcl-2 has been shown to localize to the nucleus and to induce apoptosis by preventing the transport of transcription factors such as NF kappa B into the nucleus. Apoptosis of producer cells during vector packaging and potential disruption of apoptotic pathways may explain why the titre of LV.CMV.Bcl-2 was so low in this study. Although we did not specifically investigate the influence of Bcl-2 over-expression on the mitochondria, further investigation may lead to insights into the effect on energy production and apoptosis signaling. We hypothesized that a dose dependent threshold for Bcl-2 activity exists, whereby lower activity has an anti-apoptotic effect, while higher activity results in apoptosis. Reduction in activity was achieved in this study using a tissue specific promoter, which is similar to an approach taken to package Factor VIII into a lentiviral construct [20]. 

Repression of toxic or apoptotic transgene expression during vector production may also be achieved by using the Transgene Repression in vector Production (TRiP) system. TRiP utilizes a translational block of the transgene to restrict expression, thereby enhancing lentivirus titre [19]. This transgene-specific toxicity was shown to be ameliorated in a vector-independent manner. While mechanistically unique, the concept of limiting transgene expression during packaging to improve vector yield is still the same as the approach employed in our study. 

Limitations exist in the clinical application of lentiviral vectors. Lentiviral vectors are highly efficient at integrating into the host cell genome. As a result, it is possible that gene dysregulation could occur at the site of integration which could instigate activation of oncogenes or inactivation of tumour suppressor genes [21]. Therefore, when progressing to clinical applications, it is more feasible to select an adeno-associated virus (AAV) vector which has lower immune responses and a higher safety profile [22,23]. 

AAV vectors allow cell-specific delivery of transgene to target cells, due to capsid interactions with cell receptors. Expression levels may be regulated by both promoter optimisation as well as capsid selection. Some AAV capsids exhibit superior cell entry but modest gene expression levels, due to possibly differences in intracellular trafficking [24]. These variants would be ideal to use as the gene delivery vehicle if Bcl-2 were to be used as the therapeutic transgene.

While these results are promising, Bcl-2 may have limited utility as a cardioprotective agent for cancer-therapy-induced cardiac toxicity. The therapeutic range is narrow, with a risk of switching from anti-apoptotic to pro-apoptotic, despite strategies to strictly regulate expression below a beneficial threshold. Nevertheless, our study offers proof of concept that it is possible to protect against anthracycline toxicity using an anti-apoptotic gene.

## 5. Conclusions

By controlling leaky Bcl-2 expression during transfection, lentivirus titre was improved during vector packaging. This resulted in production of functional vector which could induce an anti-apoptotic cardioprotection in NRVMs injured with doxorubicin.

## Figures and Tables

**Table 1:** Primer sequences for amplification of Bcl-2 and GAPDH.

Target	Primer	Sequence
Bcl-2	Forward	5′-GAGGATTGTGGCCTTCTTTG-3′
	Reverse	5′-ACAGTTCCACAAAGGCATCC-3′
GAPDH	Forward	5′-ACCCACTCCTCCACCTTTG-3′
	Reverse	5′-CTCTTGTGCTTGCTGGG-3′

## Data Availability

The data that support the findings of this study are available from the corresponding author upon request.
